# No difference in between-country variability in use of newly approved orphan and non- orphan medicinal products - a pilot study

**DOI:** 10.1186/1750-1172-4-27

**Published:** 2009-12-14

**Authors:** Pieter Stolk, Harald E Heemstra, Hubert GM Leufkens, Brigitte Bloechl-Daum, Eibert R Heerdink

**Affiliations:** 1 Division of Pharmacoepidemiology and Pharmacotherapy, Utrecht Institute for pharmaceutical Sciences, Utrecht University, PO Box 80082, 3508 TB, Utrecht, The Netherlands; 2 Department of Clinical Pharmacology, Medical University Vienna, Austria

## Abstract

**Background:**

Regulators and payers have to strike a balance between the needs of the patient and the optimal allocation of resources. Drugs indicated for rare diseases (orphan medicines) are a special group in this context because of their often high per unit costs. Our objective in this pilot study was to determine, for drugs used in an outpatient setting, how utilisation of centrally authorised drugs varies between countries across a selection of EU member states.

**Methods:**

We randomly selected five orphan medicines and nine other drugs that were centrally authorised in the European Union between January 2000 and November 2006. We compared utilisation of these drugs in six European Union member states: Austria, Denmark, Finland, Portugal, The Netherlands, and Sweden. Utilisation data were expressed as Defined Daily Doses per 1000 persons per year. Variability in use across countries was determined by calculating the relative standard deviation for the utilisation rates of individual drugs across countries.

**Results:**

No association between orphan medicine status and variability in use across countries was found (P = 0.52). Drugs with an orphan medicine status were more expensive and had a higher innovation score than drugs without an orphan medicine status.

**Conclusions:**

The results show that the variability in use of orphan medicines in the different health care systems of the European Union appears to be comparable to the other newly authorised drugs that were included in the analysis. This means that, although strong heterogeneity in access may exist, this heterogeneity is not specific for drugs with an orphan status.

## Background

In every health care system regulators and third party payers have to strike a balance between the needs of the individual patient and the optimal allocation of resources. For new pharmaceuticals, national regulations and traditions are important determinants for how individual drugs are embedded in the system. This embedding process is subject to multiple factors that require a number of decisions. For example, payers have to decide whether or not the drug should be reimbursed and under what conditions. For most drugs, the outcome of this process is determined by the interactions between an internationally organised supply side, represented by pharmaceutical companies, and national regulators who manage the demand side within their health care system. The positions of each party are determined by a large number of parameters, such as specific country characteristics, the economic position of the pharmaceutical company, political developments, or scientific discoveries.

Studying the variation in uptake of drugs across health care systems can provide information on how the outcome of the embedding process in the form of access to, and use of, new therapies differs from one country to another. Drugs developed specifically for the treatment of rare diseases ('orphan medicines') are a group of special interest in this context because of their often high per unit costs and for usually not being able to fulfil the standard cost-effectiveness criteria that are used in reimbursement decisions [1,2]. This may lead to drugs that are indicated for the treatment of rare diseases being more susceptible to variation in access and use than other drugs, thus leading to a stronger heterogeneity in use. The possibly specific problems with the availability of drugs for rare diseases, has been high on the agenda of organisations such as the European Organisation for Rare Diseases (Eurordis) for several years [3,4].

At the European Union (EU) level, the European Medicines Agency (EMEA) provides a centralised market authorisation procedure for new medicinal products, with a harmonised Summary of Product Characteristics (SmPC) for the whole European Union (EU) since 1995. Therefore, the EMEA centralised procedure allows the comparison of the use of drugs in different EU health care systems for drugs of which the quality, safety and efficacy were assessed by one and the same institution.

Currently, the centralised procedure is mandatory for biotechnology drugs and for all medicines intended for the treatment of HIV/AIDS, cancer, diabetes, neurodegenerative diseases, autoimmune and other immune dysfunctions, and viral diseases. A centralised procedure is mandatory for orphan medicines (OMs) as well.

Our objective in this pilot study was to determine, for drugs used in an outpatient setting, how utilisation of centrally authorised drugs varies across a selection of EU member states. In particular, we were interested in determining whether drugs that have received an orphan medicine status show a higher level of variability in use than centrally authorised medicines without an orphan medicine status, and consequently are more vulnerable to heterogeneity in access and subsequent use.

## Methods

### Study population

We randomly selected fifteen drugs using SPSS version 13.0 from a list of drugs that were centrally authorised in the EU between 1 January 2000 and 30 November 2006 and could also be used in the ambulatory setting (list compiled by the authors). We randomly selected five OMs: imatinib mesilate (Glivec^®^), bosentan (Tracleer^®^), zinc acetate dihydrate (Wilzin^®^), nitisinone (Orfadin^®^) and sodium oxybate (Xyrem^®^). In addition, we randomly selected ten other/non-orphan medicines: levetiracetam (Keppra^®^), desloratidine (Aerius^®^), telmisartan/hydrochlorothiazide (Kinzalkomb^®^), emtricitabine (Emtriva^®^), apomorfine (Uprima^®^), adefovir dipivoxil (Hepsera^®^), oxybutinin (Kentera^®^), pregabalin (Lyrica^®^), efalizumab (Raptiva^®^), abacavir/lamuvidine (Kivexa^®^).

In our initial selection we also included apomorfine (Uprima^®^), but since the market authorisation for this drug was not renewed in January 2006 and because the drug was only marketed in a few of the countries in this study, we excluded it from our final analysis.

We retrieved information about the utilisation of these drugs in six European Union member states countries: Austria, Denmark, Finland, Portugal, The Netherlands, and Sweden. These countries represent a selection of EU member states from different regions and with different health care systems, and for which drug utilisation data for all drugs included in the analysis was available.

### Utilisation rates of drugs included in the study

We calculated drug utilisation rates as a measure of uptake in the health system. We determined utilisation rates for the year 2006, as this was the latest full calendar year in the study period.

Utilisation rates were expressed as the number of Defined Daily Doses (DDD) per 1000 inhabitants per year. The DDD is a standard dosage measure defined by the World Health Organisation [5]. If DDDs were not available for a drug we defined the DDD ourselves based on information about the average daily dose contained in the official drug label. If a drug has more than one indication, the DDD is based on its first main indication in adults.

### Variability in use

Between-country variability in use was determined by calculating the relative standard deviation (RSD) for the utilisation rates of individual drugs across countries. This measure for variability in use was calculated as follows:

This method for calculating the variability in use was used elsewhere as well [6]. Utilisation rates equal to zero were excluded from further analysis.

### Innovativeness

Innovativeness of individual drugs was rated according to a system based on an algorithm designed by Motola et al [7]. This algorithm divides newly marketed drug in five classes according to two dimensions of therapeutic innovation; availability of other treatments and the therapeutic effect. Scores for availability of other treatments ranged from 5 (drugs for diseases without recognised standard treatment) to 1 (mere technological innovation). Scores for therapeutic effect ranged from 3 (major benefit on clinical endpoints or validated surrogate endpoints) to 1 (minor or temporary benefit on some aspects of the disease). All drugs included in the study were ranked according to the two dimensions of innovation with this system. We calculated the product of both scores as numeric indicator of therapeutic innovation for all products in the study. Where available, we used a list compiled by Motola et al. which was available online [8]. For drugs for which no score was available, the innovativeness was rated independently by two of the authors (HH and PS).

### Cost

As an indicator for cost differences between drugs we sampled the cost per DDD for each drug in three of the countries in this study (Denmark, The Netherlands and Sweden). These were the countries in this study for which cost information was publicly available. Within each country, we ranked all 14 drugs according to their cost per DDD. Based on the average cost per DDD ranking in each of the countries we determined an overall cost per DDD rank for each drug. A score of 1 was assigned to the cheapest drug; a score of 14 was assigned to the most expensive drug.

## Results

The basic characteristics of the six countries included in this study are shown in Table 1. This Table also provides information about the data sources from which the utilisation data in this study was retrieved.

**Table 1:** Characteristics of countries. Source: OECD Health Data 2007 and EuroStat. All data for 2006 unless otherwise indicated.

	Austria	Finland	Netherlands	Sweden	Denmark	Portugal
Population (millions) (2006)	8.27	5.26	16.33	9.05	5.43	10.57
Population aged 65 and over (%)	16.3	15.9	13.8	17.3	15.1	17.0
GDP per capita (US Dollar)	34 394	30 911	35 112	32 111	34 110	20 030
Health expenditure as a share of GDP (%)	10.2	7.5	9.2	9.1	9.1	10.2
Pharmaceutical spending per capita (US Dollar PPP)	409	380	318	351	276	445
Pharmaceutical spending (% of GDP)	1.2	1.2	1.0	1.1	0.8	2.2
Automatic reimbursement for all marketed products^a^	No	No	No	No	No	No
Pharmacoeconomic assessment required for reimbursement*	Possibly	Yes	Only for innovative medicines	Cost-effectiveness is one a criterion which determines reimbursement eligibility	Not mandatory, sometimes used to justify a higher price	Only for innovative medicines
Utilisation data source	Claims data of the Austrian sickness fund (PEGASUS-SV)	Ambulatory and hospital from the Finish National Agency for Medicines http://www.nam.fi	Ambulatory data from the GIP database http://www.gipdatabank.nl	Ambulatory data from Apoteket	Ambulatory and hospital data from the Danish Medicines Agency http://www.medstat.dk	Dispensing data of ambulatory care nationwide and IMS for hospital data (SIC-MED system)
Data type	Reimbursement	Dispensing	Reimbursement	Dispensing	Dispensing	Dispensing

^a^Pharmaceutical Pricing and Reimbursement Information (PPRI) report, the PPRI Network 2008. See http://ppri.oebig.at/

GDP: Gross Domestic Product

Table 2 provides an overview of the fourteen drugs included in the final analysis. For each drug, the date of EU market authorisation, the indication, the DDD used in the analysis, the innovation score, the cost per DDD ranking, and the RSD for the utilisation rate across the countries are reported.

**Table 2:** Overview of the drugs included in the study

Active substance	Market authorisation date	Indication	DDD	Innovation score	Cost per DDD ranking	RSD for utilisation	Number of countries with > 0 utilisation
**With orphan status**							
imatinib	11 Nov 2001	Chronic myeloid leukaemia, acute lymphoblastic leukaemia, myelodysplastic/myeloproliferative diseases, advanced hypereosinophilic syndrome, chronic eosinophilic leukaemia, metastatic malignant gastrointestinal stromal tumours, dermatofibrosarcoma protuberans	400 mg^b^	12	12	7.2%	5
bosentan	15 May 2002	Pulmonary arterial hypertension	0.25 g	6^c^	14	37.1%	6
zinc acetate dehydrate	13 Oct 2004	Wilson's disease.	0.15 g	15	6	44.9%	4
nitisinone	21 Feb 2005	Hereditary tyrosinemia type 1	20 mg	15^c^	13	112.1%	4
sodium oxybate	13 Oct 2005	Cataplexy in narcolepsy patients.	7.5 g	10^c^	11	127.6%	3

**Without orphan status**							
levetiracetam	29 Sep 2000	Epilepsy	1.5 g	8	5	29.1%	6
telmisartan/hydrochlorothiazide	19 Apr 2002	Essential hypertension.	65 mg^a^	9^c^	2	110.0%	4
emtricitabine	24 Oct 2003	HIV-1 infected adults and children in combination with other antiretroviral agents.	0.2 g	9^c^	7	142.5%	5
adefovir dipivoxil	6 Mar 2003	Chronic Hepatitis B	10 mg	12	9	89.2%	5
oxybutinin	26 Feb 2004	Symptoms of urge incontinence and/or increased urinary frequency.	15 mg	2	3	90.5%	6
pregabalin	6 Jul 2004	Peripheral and central neuropathic pain	0.3 g	10^c^	4	68.9%	6
efalizumab	20 Sep 2004	Moderate to severe chronic plaque psoriasis	10 mg	8^c^	10	53.5%	5
abacavir/lamuvidine	17 Dec 2004	Combination therapy for Human Immunodeficiency Virus (HIV).	0.9 g^a^	9^c^	8	109.9%	5
desloratidine	21 Sep 2000	Allergic rhinitis and chronic idiopathic urticaria	5 mg	3	1	41.1%	6

^a ^Combination preparation, DDD determined according to WHO guidelines. ^b ^DDD determined by authors based on literature, not WHO-CC. ^c ^Innovativeness score determined by authors based on algorithm by Motola et al.

RSD: Relative Standard Deviation, DDD: Defined Daily Dose

**Figure 1:**
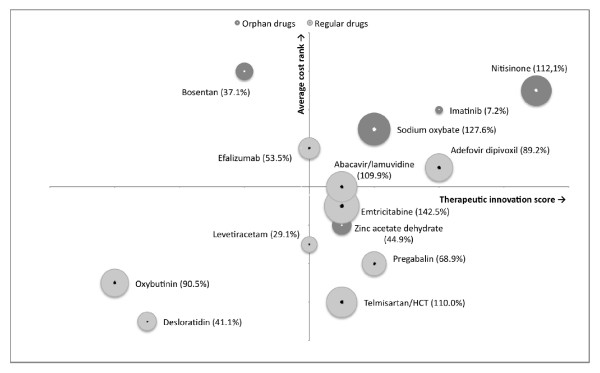
**Innovativeness versus cost matrix for centrally authorised drugs with and without orphan status**. X-axis depicts therapeutic innovativeness, while the Y-axis shows average cost per DDD rank. The size of the bubbles describes the variability in use across the countries in the study. Exact locations in the matrix are shown by dots within the bubbles. No statistically significant difference in variation in use was found (P = 0.52).

In Figure 1 we have displayed the relationship between, cost, innovativeness and variability in use for each of the drugs. The innovativeness score for each drug is depicted on the x-axis, the y-axis shows the cost ranking, while the bubble sizes denotes variability in use. The dark gray bubbles are orphan medicines, the light gray bubbles are drugs without an orphan medicine status. The Figure can be divided in roughly four quadrants. The lower left quadrant contains drugs with a low score for innovativeness and low treatment costs. The upper right quadrant contains drugs with a high innovativeness score and high treatment costs. As the Figure shows, drugs with an orphan medicine status are, in general, more expensive and have a higher innovation score than drugs without an orphan medicine status. An independent samples t-test (alpha = 0.05; SPSS version 13.0) showed that there is no association between variability in use and orphan medicine status (P = 0.52).

## Conclusions

The results from this pilot study show that the variability in use of orphan medicines in the different health care systems of the European Union appears to be comparable to the other newly authorised drugs that were included in the analysis. This means that, although strong heterogeneity in access may exist, this heterogeneity is not specific for orphan medicines. Therefore, orphan medicines may not constitute a 'special' group in this respect; heterogeneity may be an intrinsic aspect of the drug market in the European Union as a whole.

Orphan medicines rated generally higher on our innovativeness rating system than drugs without an orphan medicine status. The requirements for an orphan status are strongly congruent with the requirements for an 'Important' innovation in the model of Motola et al. One of the requirements for a drug to be eligible for an orphan medicine status is that "there exists no satisfactory method of diagnosis, prevention or treatment of the condition in question that has been authorised in the Community or, if such method exists, that the medicinal product will be of significant benefit to those affected by that condition [9]." Therefore, the concept of innovativeness in this study should be understood within the constraints of the definition used.

The outcome of the embedding process of a drug in clinical practice, and the ensuing utilisation, is the result of the interactions between drug manufacturers and regulators. Sources of variability exist at each of the stages of the embedding process. In the first place, the structural characteristics of each health care system vary significantly in, amongst others, population, the legal framework, budgets, prescribing habits by physicians and disease prevalence. Some of these sources of variability are described in Table 1.

Second, the role of the companies and regulators differs from case to case. For example, the international scope of drug manufacturers may lead to strategic decisions at the company level not to spend resources on marketing and reimbursement in a particular market. Also, the intricacies of price setting systems may make it beneficial to enter national markets in a particular order. Furthermore, for smaller companies, it may not be possible to market a drug product in all countries at the same time and a company may decide to introduce the product on the European market in phases.

Third, regulators in each country face unique inputs that may influence their assessment of an individual drug, such as constraints in (drug) budgets, the influence of patient organisations, or political pressure.

The interactions between different regulators and companies in the context of the structural characteristics of each country can lead to a wide variety of outcomes, which are designed to influence actual use in a different way. As the results from this pilot study indicate neither cost per DDD, nor innovativeness or an orphan status influences the variability in use between the countries in the study.

Certain limitations apply to this pilot study. Given the small sample sizes, it is very difficult to disentangle the specific contributions of innovativeness and cost to the variability in use. In addition, when including per unit treatment costs in our analysis, we used a method of ranking drugs according to their cost per DDD which only included costs in three countries, thus disregarding costs in the three other countries in the study. However, it is unlikely that relative costs in the other three countries in the study would differ significantly. We have no reason to believe that the ranking based on relative cost per DDD would show large variations when these other countries would have been included as well.

Furthermore, we only looked at the national level in this study and did not take regional variation into consideration. Access to, and use of, drugs may show large regional variability, for example, depending on policy and budget considerations of individual hospitals or insurance companies [10].

Another limitation is that we did not include the indication of the drugs into our analysis. For some of the primary indications of drugs included in this study the prevalence may vary across countries, thus leading to an overestimation of the RSD measure for the utilisation of these drugs. Also, for several orphan medicines, such as zinc acetate, compounded alternatives may be available, which would make our measurement of the utilisation of this drug an underestimate in some countries. Furthermore, for some of the drugs alternative treatments may be available.

The data sources used in this study consisted of reimbursement and dispensing data. Therefore, our results may have been influenced by differences between reimbursement and utilisation within countries or by differences by the location of data collection (public pharmacy, hospital pharmacy or both). As this limitation is an inherent characteristic of the data, we have indicated the source of our data in Table 2. This also indicates that there is a need for a harmonised method of data collection on drug utilisation within Europe.

Finally, the countries included constitute a selection of smaller and medium sized EU member states. Results from this pilot study should therefore be extrapolated to other EU member states with caution; regulatory systems and GDP may vary significantly from the countries included in the study. For two groups it is especially important to note their absence. Firstly, none of the large member states such as Germany, France and the UK are included in the analysis. Furthermore, countries that joined the EU on or after 1 May 2004 are also not included. The Eurordis survey on orphan medicines that was mentioned earlier^4^, specifically mentions newer EU countries as countries with potentially low availability of orphan medicines. These countries may well have specific challenges with access and use of newly authorised drugs.

Overall, we believe that this study provides interesting preliminary data that warrants future investigations in which the methodological constraints of this study can be addressed.

In conclusion, we found that orphan medicines show no larger variability in use than drugs without an orphan medicine status. Therefore, heterogeneity in variability may be a feature of the drug market in the EU in general, and not restricted to one class of drugs. Future studies looking at access issues should also take the actual utilisation into account for a comprehensive assessment of this topic.

## Competing interests

The authors declare that they have no competing interests.

## Authors' contributions

PS and HH participated in the design of the study, data collection, data analysis and writing of the manuscript. BB-D participated in the data collection and the study design. EH and HL participated in the study design and the analysis of the study. All authors read and approved the final manuscript.
